# Steroid receptors and their regulation in avian extraembryonic membranes provide a novel substrate for hormone mediated maternal effects

**DOI:** 10.1038/s41598-019-48001-x

**Published:** 2019-08-08

**Authors:** Neeraj Kumar, Anja Lohrentz, Manfred Gahr, Ton G. G. Groothuis

**Affiliations:** 1 Behavioural Biology, Groningen Institute for Evolutionary Life Sciences, University of Groningen, Groningen, The Netherlands; 2 Behavioural Neurobiology, Max Planck Institute for Ornithology, Seewiesen, Germany

**Keywords:** Physiology, Animal physiology

## Abstract

Exposure of the vertebrate embryo to maternal hormones can have long-lasting effects on its phenotype, which has been studied extensively by experimentally manipulating maternal steroids, mostly androgens, in bird eggs. Yet, there is a severe lack of understanding of how and when these effects are actually mediated, hampering both underlying proximate and ultimate explanations. Here we report a novel finding that the embryo expresses androgen receptor (AR) and estrogen receptor (ERα) mRNA in its extraembryonic membranes (EMs) as early as before its own hormone production starts, suggesting a novel substrate for action of maternal hormones on the offspring. We also report the first experimental evidence for steroid receptor regulation in the avian embryo in response to yolk steroid levels: the level of AR is dependent on yolk androgen levels only in the EMs but not in body tissues, suggesting embryonic adaptation to maternal hormones. The results also solve the problem of uptake of lipophilic steroids from the yolk, why they affect multiple traits, and how they could mediate maternal effects without affecting embryonic sexual differentiation.

## Introduction

In many animal taxa, including vertebrates, the embryo is exposed to maternal hormones, which can have long-lasting effects on its phenotype (fish^1^, reptiles^2^, birds^3–5^, mammals^6,7^). Several studies have injected steroids, mostly androgens, into bird eggs, the most widely used model, mimicking variation in maternal yolk deposition and finding a wide array of effects on the offspring phenotype^3–5,8^. The mechanisms underlying such effects are largely ignored, hampering further progress in this prevalent field of research^9^. In order to be functional, the androgens must reach the embryonic tissues and those tissues must have androgen receptors (AR). However, very early in incubation, yolk androgens seem to be rapidly metabolized to inactive forms by the embryo^10–12^. Moreover, in spite of being polar, steroid hormones are lipophilic and do not easily dissolve in water. Therefore, it remains an enigma how the embryo is able to take up these hormones from the lipid rich yolk into its watery circulation for their transport to body tissues where they can exert their effects.

**Figure 1:**
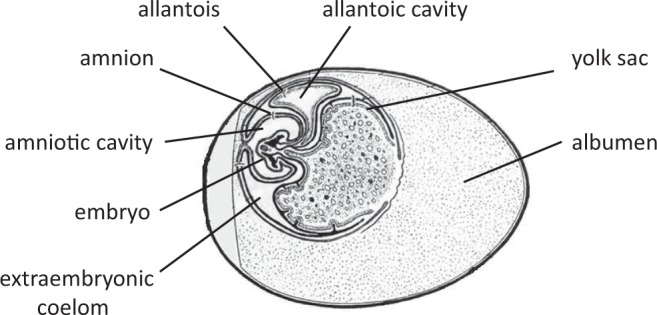
A schematic representation of development of chicken embryo and its extraembryonic membranes after five days of egg incubation (redrawn with modifications after Patten, 1920)^45^.

We tested the hypothesis that the embryo expresses AR and/or estrogen receptors (ERα, as alpha is the most commonly studied isoform in bird species) in the extraembryonic membranes (EMs) where maternal hormones could act without the need to reach to body tissues. The embryo produces EMs – yolk sac, amnion, chorion, and allantois, that support embryo’s nutrition, physical protection, respiration, and excretion^13^, having similar functions as the fetal placenta in mammals. The EMs are at the immediate interface of the maternal egg yolk containing the maternal hormones and the circulation of developing embryo (Fig. 1), making these a potential candidate for mediating effects of maternal hormones on the embryo.

It has been shown earlier that yolk androgens can affect AR expression in the brain of the young chick^14^. However, it remains unknown to what extent such receptors are present and influenced by yolk hormones at the interface of the yolk and embryonic circulation, already early in embryonic development, before these hormones are metabolized during the first days of incubation. Therefore, we also tested the hypothesis whether the androgen treatment could induce changes in AR and/or ERα expression in embryonic tissues. If so, this would indicate that the embryo is an active player in the translation of the maternal signal.

We assessed the effect of elevated yolk testosterone (T) and, in other eggs, androstenedione (A4), within the physiological range of the species on AR and ERα expression in the EMs and in embryonic body tissues (the head and the decapitated body) analysed by quantitative PCR (qPCR), using chicken eggs incubated for five days. This time-period was chosen because the gonadal differentiation^15,16^ and the surge of the endogenous steroid production^17^ in the chicken embryo starts only after this period.

## Results

**Figure 2:**
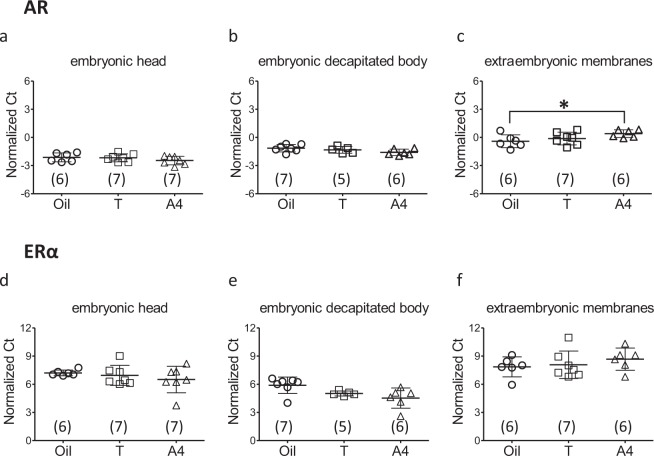
qPCR analysis for receptor mRNA expression. The comparison of androgen (AR, panels (a–c) and estrogen (ERα, panels d–f) receptor mRNA expression plotted as normalized Ct values (individual data plotted together with mean and standard deviation) in embryonic head, decapitated body, and extraembryonic membrane tissues from chicken eggs injected with T, or A4, or oil as a control, followed by incubation for five days. The receptor expression levels are inversely related to the normalized Ct values. The numbers in parentheses represent sample sizes, *p < 0.05.

AR mRNA was expressed in all three embryonic fractions: head, decapitated body, and EMs (Fig. 2a–c). It shall be noted that the receptor expression levels are inversely related to the normalized threshold cycle (Ct) values of the qPCR procedure. There was no significant overall effect of the egg treatment on AR expression levels (F_2,48_ = 0.011, p = 0.989), but there was a significant interaction effect between egg treatment and embryonic tissue (F_4,48_ = 3.266, p = 0.019). Tukey’s post-hoc comparisons revealed a significant downregulation of AR expression under A4 treatment only in the EMs (p = 0.016, Table 1).

**Table 1:** Tukey’s post-hoc comparisons for the effect of egg treatment (A4, T, or Oil) on AR expression in embryonic tissues (head, decapitated body, and extraembryonic membranes).

	Estimate	Standard error	p-value
**Embryonic head**			
A4 vs T	−0.290	0.261	0.511
A4 vs Oil	−0.338	0.272	0.433
T vs Oil	−0.048	0.272	0.983
**Embryonic decapitated body**			
A4 vs T	−0.258	0.298	0.664
A4 vs Oil	−0.437	0.272	0.252
T vs Oil	−0.179	0.288	0.809
**Extraembryonic membranes**			
A4 vs T	0.517	0.272	0.148
A4 vs Oil	0.813	0.282	**0.016**
T vs Oil	0.296	0.272	0.525

ERα mRNA was also expressed in all three embryonic fractions, but to much lower levels than AR (Fig. 2d–f). There was no significant overall effect of the egg treatment on ERα expression levels (F_2,48_ = 0.754, p = 0.476), and neither was any significant interaction effect between egg treatment and embryonic tissue (F_4,48_ = 1.737, p = 0.157).

## Discussion

It is generally assumed that avian maternal hormones in the egg can be functional only if they reach embryonic body tissues. Here we report that both ARs and ERs are expressed in avian EMs (Fig. 2) as early as approximately one-fourth of the entire egg incubation period until hatching, before the embryo’s own hormone production starts^15–17^, opening up a novel, potential pathway for hormone mediated maternal effects. The EMs, particularly the yolk sac, provide potent sites for embryonic contact with yolk contents due to their relatively larger surface area and denser blood vessel networks, compared to the embryonic body tissues (Fig. 1). Furthermore, we found that AR expression is dependent on yolk A4 levels only in the EMs, suggesting embryonic adaptation to its exposure to maternal androgens in the egg as the EMs are right at the interface of maternal yolk environment and embryonic circulation. The importance of the EMs for yolk hormones have also been shown by the fact that the EMs express enzymes that are important for regulating steroid metabolism, as found in a turtle species^18,19^. One of the steroid metabolites is etiocholanolone, which is an androgen metabolite formed during egg incubation^10,11^, and it has been suggested that etiocholanolone might influence erythropoiesis via yolk sac membrane^10^, but for which there is as yet no experimental evidence. There was no effect of T treatment on AR expression in the EMs, which could simply be due to the fact that the amount of injected T was much lower than A4.

There was no effect of increased T or A4 yolk levels on the AR and ERα mRNA expression in the embryonic body tissues (Fig. 2a,b,d,e). This suggests it is unlikely that elevated concentrations of maternal yolk androgens affect offspring phenotype by their effect on early embryonic responsiveness to its own endogenous steroids later in development (i.e. after five days of incubation). However, it should be studied further whether the androgen treatment might affect the embryonic AR and ERα receptor expression in the body tissues at later developmental stages. The levels of the ERα mRNA expression were much lower than the AR in all the embryonic fractions examined (Fig. 2).

Though several studies have previously reported steroid receptors in avian embryonic body tissues (AR^20,21^, ER^14,21–23^, progesterone receptor (PR)^24,25^, glucocorticoid receptor (GR)^26^), the data on receptors in the EMs are scarce. Two of these membranes, chorion and allantois, in combination form a tissue lining at the inner surface of the eggshell, known as the chorioallantoic membrane. Chorioallantoic membrane tissue was found to express AR^27^, ER^28^, and PR^25^ in 8 to18 days old chicken embryos, chicken embryos partly cultured in petri dishes^29^, as well as in reptiles^27,30^. However, the chorioallantoic membrane starts to develop only after day 4 and at a very slow rate^31^, contributing less than 5% to the total EMs dry weight by day 5^32^. This indicates that the high receptor expression that we found is very likely to be localized in the yolk sac membrane itself and should be further verified. The yolk sac membrane is in a much better position than the chorioallantoic membrane for translating yolk hormones to the embryo as the chorioallantoic membrane does not have direct access to the yolk and hence maternal hormones. The mammalian fetal placenta, an equivalent of part of the avian EMs, has also been found to express AR^33^, ER^34^, and GR^35–37^, mediating effects of maternal condition, however their presence has always been measured at much later stages of embryonic development. That is typically at the time of delivery with only one exception of about 55% completion of fetal development^34^ while we measured the receptors already at 24% of the total embryonic development period.

Another long-standing question in the field is how the gonadal sex-steroids in the egg mediate maternal effects without interfering with embryonic sexual differentiation processes^38^. One potential explanation is very early embryonic metabolism of maternal steroids, i.e. prior to the critical time-window for sexual differentiation^11,39^. Our proposed mechanism, activating ARs in the EM very early, provides an additional potential solution to this problem as we postulate that maternal steroids need not reach embryonic body tissues to mediate maternal effects. Furthermore, maternal hormones could induce receptor mediated transcription long before organs that undergo sexual differentiation, such as the hypothalamus, are developed. Additionally, the activation of the receptors in the EMs so early in the process of building a new organism and its expression not being limited to specific brain or other tissues might also explain the wide array of maternal hormone effects observed in the literature.

The location of these receptors may explain how the lipophilic hormones in the yolk that would be difficult to extract and taken up in the embryo’s circulation, can affect the embryo. However, it remains to explore further what kind of molecular and physiological effects are elicited via AR activation in the EMs. Finally, the receptor downregulation caused by increased yolk A4 levels indicates that the embryo can to some extent negate potential effects of elevated hormone exposure, suggesting that the embryo is not simply a passive receiver of the mother’s signals but may play its own role in mother-offspring conflict^40–43^.

## Methods

### Animal ethics

This study used five days old chicken embryos, which does not require an ethical license or approval from an animal experimentation committee.

### Experimental design

Fertilized unincubated chicken eggs of Lohman Brown Classic strain were randomly collected from a local chicken farm, and randomly allocated to the three weight-matched treatment groups. Each egg was injected with 100 µl of sterilized sesame oil with either 0.2 µg/ml stable isotope labelled T, or 0.58 µg/ml stable isotope labelled A4, or only oil as a control, with seven eggs per group. Stable isotope labelled androgens were used in order to track steroid metabolism using mass spectrometry as part of another study. Due to a lack of prior studies on the effect of egg hormone treatment on the embryonic receptor expression, it was not possible to make a reliable estimate of the effect size for sample size prediction. Therefore, a sample size of seven was chosen which is just above the minimum required sample size to perform statistical tests. The injected hormone values were within two standard deviations of the mean yolk hormone concentrations found in our earlier study^12^ (T = 0.74 ± 0.13 pg mg^−1^; A4 = 23.24 ± 2.20 pg mg^−1^; means ± s.d.). The eggs were subsequently incubated for five days at 37 °C at a relative humidity of 60% in an incubator (Brinsea Ova-Easy Advance). At the end of five days of incubation each individual embryo (of either sex) was separated into three fractions: embryonic head, decapitated body, and EMs, which were frozen at −80 °C until AR and ERα mRNA expression analysis took place.

### AR and ERα mRNA expression analysis

The receptor mRNA expression was analysed by qPCR by a technician blind to the treatment groups. We started with seven different embryos for each of the three treatments (T, A4, oil) per tissue type (embryonic head, decapitated body, and EMs). Tissue was homogenized (Tissue Ruptor, Qiagen). Total RNA was isolated from deep frozen tissue according to the kit instructions (RNeasy Mini kit, Qiagen). RNA Quality was measured using Bioanalyzer 2100 (Agilent) and quantified using Nanodrop (Peqlab), of which the descriptive statistics (average, standard deviation, and range per treatment group for each tissue type) is provided in Supplementary Table 1. Out of 63 samples, 6 did not yield sufficient RNA and thus could not be analysed further. For the remaining 57 samples, cDNA was synthesized from total RNA according to the kit instructions (SuperScriptIII, Invitrogen) and was diluted 1:5 in water as template in qPCR experiment. Power SYBr green qPCR mastermix was used from Thermo Fisher Scientific. PCR protocol included the following steps: denaturation at 95 °C for 30 seconds; annealing at 60 °C for 60 seconds; elongation at 72 °C for 30 seconds; cycle repeat for 35 times. The primers used are listed in Table 2. The primer efficiency was tested by a dilution series and their amplicons were sequenced (MWG Operon Eurofins Genomics). The Ct values were normalized using GenEx6 software for the efficiency of primers, sample amount (RNA input into cDNA synthesis), qPCR repeats (duplicates), and for two reference genes – hydroxymethylbilane synthase (HMBS) and tyrosine 3-monooxygenase/tryptophan 5-monooxygenase (YWHAZ). There was no significant effect of egg treatment (F_2,15.5_ = 0.686, p = 0.518 for HMBS; F_2,16.8_ = 1.890, p = 0.182 for YWHAZ) or interaction effect between egg treatment and embryonic tissue (F_4, 30.7_ = 1.319, p = 0.285 for HMBS; F_4,31.5_ = 0.291, p = 0.881 for YWHAZ) for either of the reference genes (data is provided in Supplementary Table 2). All samples were run on one plate. The intra-assay coefficient of variation was 7.3% for AR and 5.5% for ERα.

**Table 2:** Primer pairs used for qPCR.

Gene	Forward primer	Reverse primer
AR	gatggcctgaagaaccagaa	gaaatgatggccgagatca
ERα	ttcaaggggaggaatttgtg	tgtccagaacacggtggata
HMBS	cctcagctagaattcagggatatt	gattctcccagcccattctc
YWHAZ	gttgctgctggagatgacaa	atctgatcggatgtgttggc

### Statistics

The data were analysed by linear mixed model using IBM-SPSS (version 23) and R^44^ (version 3.5.3) software. The normalized Ct values were analysed for each receptor gene (AR or ERα) and reference gene (HMBS or YWHAZ) by taking the Ct value as a dependent variable, egg treatment (three levels: oil, T, and A4) and embryonic tissue (three levels: head, decapitated body, and EMs) as well as their interaction as fixed factors, and the embryo identity as a random factor, followed by Tukey’s post-hoc tests for multiple comparisons.

## Supplementary information

Supplementary information

Dataset 1

## Data Availability

The datasets supporting this article are provided in the supplementary material.
