# Supplementary material for: Steroid receptors and their regulation in avian extraembryonic membranes provide a novel substrate for hormone mediated maternal effects
Source: Sci Rep. 2019 Aug 8;9:11501. doi: 10.1038/s41598-019-48001-x (PMC6687743; doi:10.1038/s41598-019-48001-x)
Supplement: Supplementary file 1 — Supplementary information [file 41598_2019_48001_MOESM1_ESM.pdf]

Title

Steroid receptors and their regulation in avian extra-embryonic membranes provide a novel substrate for hormone-mediated maternal effects

Authors

Neeraj Kumar[1,2*], Anja Lohrentz[2], Manfred Gahr[2], Ton G.G. Groothuis[1]

[1]*Behavioural Biology, Groningen Institute for Evolutionary Life Sciences, University of Groningen, the Netherlands*

[2]*Behavioural Neurobiology, Max Planck Institute for Ornithology, Seewiesen, Germany*

*To whom correspondence should be addressed.

e-mail: neeraj.bioscience@gmail.com

Supplementary information

Supplementary Table 1. The descriptive statistics of RNA quality and quantification parameters.

	**RNA Integrity Number (RIN)**			**RNA concentration (ng/µl)**			**A260/A280**		
Tissue	*average*	*SD*	*range*	*average*	*SD*	*range*	*average*	*SD*	*range*
**Treatment: A4**									
1	9.66	0.15	9.5 - 9.9	1219.94	241.19	918.11 - 1616.32	2.06	0.01	2.05 - 2.09
2	7.55	1.29	5.1 - 8.6	158.18	124.31	4.85 - 389.74	2.07	0.15	1.96 - 2.41
3	7.97	0.43	7.3 - 8.5	58.50	28.50	8.81 - 100.93	2.15	0.14	2.06 - 2.46
**Treatment: T**									
1	9.47	0.24	9.2 - 9.8	1333.72	222.72	1023.79 - 1552.74	2.07	0.01	2.06 - 2.08
2	8.93	0.42	8.1 - 9.4	148.13	93.95	11.58 - 240.25	2.09	0.17	1.93 - 2.36
3	8.49	0.32	8.0 - 8.8	109.87	32.44	69.27 - 144.51	2.06	0.03	2.04 - 2.11
**Treatment: Oil**									
1	9.33	0.36	8.6 - 9.7	1130.44	179.16	870.67 - 1378.13	2.06	0.01	2.05 - 2.07
2	8.03	0.72	6.9 - 9.1	143.28	20.26	112.63 - 168.92	2.02	0.02	1.99 - 2.04
3	8.80	0.45	8.1 - 9.3	75.90	39.59	7.77 - 130.71	2.08	0.07	1.99 - 2.21

Tissue: 1 = embryonic head, 2 = embryonic decapitated body, 3 = extra-embryonic membranes

SD = standard deviation

Supplementary Table 2. The Ct values for the two reference genes (HMBS and YWHAZ) which were used to normalize the AR and ERα receptor expression.

Egg Treatment	HMBS			YWHAZ		
	head	*decapitated body*	*EMs*	*head*	*decapitated body*	*EMs*
1	29.17	29.10	32.85	28.00	29.82	37.79
1	28.64	27.81	26.66	27.97	27.09	30.22
1	28.31	29.14	27.45	27.40	28.18	31.11
1	28.04	28.23	28.33	27.20	27.58	31.89
1	28.20	28.78	27.11	27.28	27.50	30.10
1	28.40	28.30	27.13	27.73	26.53	29.99
1	28.46	NA	26.99	27.88	NA	29.72
2	28.39	28.46	26.15	27.07	27.60	28.74
2	27.97	27.69	26.96	27.52	26.72	29.27
2	28.56	27.50	28.14	26.98	26.13	30.03
2	27.54	27.61	27.46	27.03	27.60	30.90
2	28.18	28.21	27.76	27.17	27.05	30.19
2	28.84	NA	28.47	28.26	NA	30.06
2	28.32	NA	27.90	27.36	NA	30.47
3	28.83	27.39	26.94	27.37	26.45	28.06
3	28.46	28.74	27.62	27.57	27.43	29.53
3	28.79	28.19	26.93	27.58	27.51	29.42
3	27.99	28.04	27.71	27.00	27.65	30.89
3	29.10	29.45	27.65	27.60	28.69	31.47
3	28.43	28.28	27.52	27.09	27.48	30.64
3	NA	28.06	NA		27.39	NA

Treatment: 1 = A4, 2 = T, 3 = Oil

NA = data not available
